# Affective and enjoyment responses to 12 weeks of high intensity interval training and moderate continuous training in adults with Crohn’s disease

**DOI:** 10.1371/journal.pone.0222060

**Published:** 2019-09-20

**Authors:** Lindsay Bottoms, Dean Leighton, Roger Carpenter, Simon Anderson, Louise Langmead, John Ramage, James Faulkner, Elizabeth Coleman, Caroline Fairhurst, Michael Seed, Garry Tew

**Affiliations:** 1 Department of Psychology and Sports Science, University of Hertfordshire, Life and Medical Sciences, Hatfield, United Kingdom; 2 Centre for Immunobiology, Queen Mary University of London, Whitechapel, London; 3 School of Health, Sport and Bioscience, University of East London, Stratford Campus, London, United Kingdom; 4 Guy’s and St Thomas’ NHS Foundation Trust, London, United Kingdom; 5 Digestive Diseases Clinical Academic Unit, Barts and the London NHS Trust, London, United Kingdom; 6 Hampshire Hospitals NHS Foundation Trust, Hampshire, United Kingdom; 7 Department of Sport, Exercise and Health, University of Winchester, Winchester, United Kingdom; 8 York Trials Unit, University of York, Heslington, York; 9 Department of Sport, Exercise and Rehabilitation, Northumbria University, Newcastle, United Kingdom; University of Alabama at Birmingham, UNITED STATES

## Abstract

The aim was to undertake secondary data analysis from a three-arm randomised feasibility trial of high intensity interval training (HIIT), moderate intensity continuous training (MICT), and usual care control in adults with Crohn’s disease (CD; n = 36), with a primary focus on exploring affective and enjoyment responses. Twenty-five participants with quiescent or mildly-active CD were randomised to one of the two exercise groups: HIIT (n = 13) and MICT (n = 12). Both groups were offered thrice weekly sessions for 12 weeks. MICT consisted of cycling for 30 minutes at 35% peak power (Wpeak), whereas HIIT involved ten 1-minute bouts at 90% Wpeak, interspersed with 1-minute bouts at 15% Wpeak. Heart rate (HR), differentiated ratings of perceived exertion for legs (RPE-L) and central (RPE-C), along with feeling state (Feeling Scale; FS) were measured at 92.5% of each session. Enjoyment was measured at the end of training using the Physical Activity Enjoyment Scale (PACES). Post-hoc exploratory analysis involved a mixed-model two-way ANOVA to compare HR, RPE-L, RPE-C and FS for the exercise sessions in weeks 1, 6 and 12 between groups. Overall, HR was greater (*p* < 0.01) during HIIT (173 ± 8 bpm) compared with MICT (128 ± 6 bpm). Similarly, RPE-L and RPE-C responses were greater overall (*p* = 0.03 and *p* = 0.03, respectively) during HIIT (5.5 ± 1.6 and 5.1 ± 1.7, respectively) compared to MICT (3.3 ± 1.5 and 2.9 ± 1.5, respectively). Overall, FS was 2.2 ± 1.9 for HIIT and 2.1 ± 1.4 for MICT with no effect of treatment group (*p* = 0.25) or time (*p* = 0.94). There was also no significant difference in PACES scores between HIIT (99.4 ± 12.9) and MICT (101.3 ± 17.4; *p* = 0.78). The findings suggest HIIT and MICT protocols elicited similar enjoyment and affect in adults with quiescent or mildly-active CD.

## Introduction

Crohn’s Disease (CD) is a type of inflammatory bowel disease which can affect a patient anywhere from the mouth to the anus. Patients often suffer with symptoms of fatigue, pain and diarrhoea [1]. Due to the inflammation of the gut wall it can also lead to malabsorption and this can lead to side effects such as low bone mineral density and loss of muscle mass [1]. Such effects can reduce quality of life in a patient [2, 3]. Other extraintestinal manifestations include large joint arthritis, uveitis, iritis, episcleritis, erythema nodosum and pyroderma gangernosum [4]. In 2006, it was observed that treatment for inflammatory bowel disease costs the United Kingdom’s National Health Service (NHS) approximately £720 million per annum [5] and with numbers of patients increasing annually the economic burden will have risen and will continue to rise.

The main goals of treatment for CD are to achieve mucosal healing and deep remission [4] and prevent the need for surgery. The treatment for CD involves an induction and maintenance regimen [6]. The choice of medication is dependent on disease severity and response to previous therapies. The most widely used drugs in CD are corticosteroids, immunosuppressants and biologics [6] with the aim to induce remission and mucosal healing and prevent the need for surgery. As mentioned, there are often extraintestinal manifestations such as fatigue, low bone mineral density and loss of muscle mass which the prescribed medications often do not treat. Therefore, exercise could be a potential adjunct therapy to help reduce these symptoms and improve quality of life, as it has been shown to help in other health conditions [7]. It therefore seems a logical progression to investigate exercise efficacy in relation to CD.

Most of the information currently known about exercise and CD is based on studies of low-moderate intensity exercise with small sample sizes, which demonstrated an improvement in quality of life without apparent adverse events [8–10]. However, results from a UK-based survey indicated that 83% of adults with IBD do not engage in levels of activity commensurate with the public health guidelines (17) of 150 minutes of moderate aerobic physical activity a week [11]. Health related research in the last decade has started to focus on the benefits of high intensity interval training (HIIT) which consists of repeated, intense exercise bouts separated by passive or active recovery and can be performed in less time for the same energy expenditure [12]. Data from 6 to 12 weeks of exercise training demonstrates similar to greater improvements in maximal oxygen uptake (${\dot{\text{V}}\text{O}}_{2\text{max}}$) with HIIT (~10–14%) compared to MICT (~7–10%)[13, 14]. This could make HIIT appealing to recommend when encouraging sedentary individuals to become more physically active. However, HIIT can produce symptoms of shortness of breath, leg pain and dramatic fatigue in comparison to MICT [15] and may therefore be less enjoyable. Currently, little information is known on HIIT in adults with CD despite it appearing as a time-efficient approach to improve cardiorespiratory fitness and cardio-metabolic health in general populations [16] and other clinical populations [17, 18]. Previous authors have suggested high-intensity exercise may potentially be detrimental to health due to possible negative side effects in IBD, such as gastrointestinal distress [8] which could exacerbate symptoms. This might be an old-fashioned reservation about high-intensity exercise and IBD as there are several examples of elite athletes who suffer with IBD such as Sir Steve Redgrave (a 5 times Olympic Champion) and Ali Jawad (a Paralympic power lifter) who have still managed to compete at an international standard. A greater understanding of the safety and efficacy of different types of exercise training is needed to support the development of evidence-based exercise guidelines and promotion strategies that are specific to CD.

Acceptability and enjoyment are also important when developing and exploring exercise training for clinical populations. Considering that the affective response may be a predictor for exercise adherence [19], it is important to prescribe exercise sessions which result in positive affective responses. More intensive exercise might result in more negative affective responses which in turn might contribute to poor exercise adherence [19]. There is a trade-off between higher intensities generally providing more cardiorespiratory fitness benefit but less favourable affective responses. HIIT becomes a viable exercise programming option because the rest intervals between intense work intervals may contribute to reduced discomfort and inducing a more positive affective response. Studies comparing affective responses of HIIT and MICT have produced mixed results [20–23] and no studies have been published in CD. Therefore, we conducted a feasibility study to determine the acceptability and potential benefits and harms of HIIT and MICT in adults with quiescent or mildly-active CD, and the feasibility of conducting a full-scale trial (see Tew *et al*. [24] for full discussion). However, as enjoyment is a potential barrier for participating in exercise we focus here on secondary analysis to explore differences in affect and enjoyment following 12 weeks of either HIIT or MICT training in CD patients.

## Materials and methods

### Experimental design

This is a secondary analysis of data collected in a three-arm, parallel-group, feasibility randomised controlled trial, which had a 12-week intervention period and follow-up assessments at 13 and 26 weeks after randomisation. Following enrolment, all participants underwent a baseline cardiopulmonary exercise test (CPET) on a cycle ergometer and were asked for their preference to a specific group allocation prior to randomisation. After baseline assessments, participants were randomly assigned to HIIT, MICT or control, with each group receiving usual NHS care. Data on affect and enjoyment was not collected in the control group, so the focus here is on data from the HIIT and MICT groups only. Further details on the trial design have been published previously [25]. Ethics approval was granted by the Camden and Kings Cross Research Ethics Committee (reference 15/LO/1804), and all participants provided written informed consent before enrolment. The trial was registered prospectively (ISRCTN13021107).

### Participants and setting

We included male and female patients between 16 and 65 years of age with a clinical diagnosis of CD. Patients had to have a stool calprotectin of <250 μg/g, stable medication (>4 weeks), and quiescent or mildly-active disease, as indicated by a Crohn’s Disease Activity Index (CDAI) of <150 or 150–219, respectively. Exclusion criteria were: contraindication to exercise testing or training [26], coexistent serious autoimmune disease (e.g. rheumatoid arthritis or systemic sclerosis), pregnant, planned pregnancy or major surgery within the first 3 months after randomisation, poor tolerability of venepuncture or inadequate access for venous blood sampling, and current participation in >90 min/week of purposeful exercise (e.g. cycling, swimming or running) or another clinical trial. Recruitment was from three hospital trusts in England: Guy’s and St Thomas’ NHS Foundation Trust, Barts Health NHS Trust, and Hampshire Hospitals NHS Foundation Trust. The exercise programmes were delivered in the exercise science facilities of the University of East London and the University of Winchester.

### Exercise intervention

Participants were invited to complete three supervised exercise sessions per week for 12 consecutive weeks, commencing the week following their baseline assessment and randomisation. Reimbursement was provided for travel expenses. All exercise was undertaken on a cycle ergometer (Lode Corival or SRM Ergometer), with each session comprising a 5-minute warm-up at 15% of peak power output (Wpeak; determined during the baseline CPET), a main conditioning phase, and then a 3-minute cool-down at 15% Wpeak. For HIIT, the conditioning phase involved ten 1-minute bouts at 90% Wpeak, interspersed with 1-minute bouts at 15% Wpeak, whereas for MICT it involved 30 minutes at 35% Wpeak. The MICT programme was selected because it has been shown to elicit a similar energy expenditure compared with the HIIT programme [27].

Differential ratings for breathlessness (RPE-C) and leg exertion (RPE-L) were assessed using Borg’s CR-10 scale [28] before exercise, immediately post, and 10-minutes post-exercise. In addition, participants were asked to rate their breathlessness and leg exertion at 2.5%, 7.5%, 42.5%, 47.5%, 92.5% and 97.5% of exercise completed. These time points were chosen to incorporate both interval and recovery periods during the HIIT protocol [21]. Participants' heart rate was also recorded using Polar heart-rate monitors at 2.5%, 42.5%, and 92.5% of exercise completed. The one item Feeling Scale (FS; [29]) was used to measure general affective valence (i.e., pleasure and displeasure). Participants were prompted at the beginning of each exercise visit with the following instructions: *“While participating in exercise*, *it is common to experience changes in mood*. *Some individuals find exercise pleasurable*, *whereas others find it to be unpleasant*. *Additionally*, *feeling may fluctuate across time*. *That is*, *one might feel good and bad a number of times during exercise*. *When asked please tell me how you feel at that current moment using the scale below”*. The feeling scale is scored on an 11-point bipolar scale ranging from -5 (very bad) to +5 (very good). The FS was administered pre-, immediately post and 10-minutes post-exercise. To assess in-task affect, the FS was also administered at 2.5%, 42.5%, and 92.5% of exercise completed. In addition, enjoyment was measured during the follow up assessment in week 13 using the Physical Activity Enjoyment Scale (PACES). Incremental cycle exercise testing to maximum volitional exertion was performed in the final sessions of weeks 4 and 8 to re-calculate Wpeak and determine if the power output of the upcoming exercise sessions needed to be changed.

### Statistical analysis

Statistical analyses were performed using SPSS v25.0 (IBM, Chicago, USA). Data are presented as means ± standard deviation (SD) and, where appropriate, individual responses are presented in a dot plot graph. Post-hoc exploratory analysis involved a mixed-model two-way ANOVA to compare exercise training data for RPE-C, RPE-L and FS on data averaged across the 3 weekly sessions at 92.5% of exercise completed (at the end of the 10^th^ interval for HIIT and 27^th^ minute for MICT) for weeks 1, 6 and 12 (time) and between groups (condition). In addition, a mixed-model two-way ANOVA to compare exercise testing data for peak power and HR at baseline and weeks 4, 8 and 12 (time) and between groups (condition). Main effects for time, main effects for condition and the interaction between time and condition were calculated and where appropriate Bonferonni post-hoc corrections for multiple comparisons were conducted. Mean differences are presented with standard error when comparisons are made. Data normality was checked using a Shapiro-Wilk test; upon moderate violation of the normality assumption, analysis of variance (ANOVA) was still conducted as the model is sufficiently robust to detect statistically significant differences between means, in terms of type 1 error [30]. There was homogeneity of variances, as assessed by Levene’s test of homogeneity of variance (*p* > 0.05). There was homogeneity of covariances, as assessed by Box's test of equality of covariance matrices (*p* >0.05). Greenhouse-Geisser correction was applied upon violation of Mauchly’s test of sphericity for ANOVAs. An independent t-test was used to assess between-group differences in PACES scores. Significance was determined by a *p* value of <0.05. Effect sizes were calculated using partial eta squared (η_p_^2^) and defined as trivial (<0.10), small (0.10–0.29), moderate (0.30–0.49), or large (≥0.50) [31].

## Results

### Participant characteristics at baseline

A total of 13 participants, of which 7 (54%) were male (mean ± SD age: 37.0 ± 11.1 yrs; body mass: 76.2 ± 13.5 kg; CDAI: 74 ± 48), were randomised to HIIT and 12 were randomised to MICT (male: n = 3, 25%; mean ± SD age: 38.5 ± 13.0 yrs; body mass: 63.8 ± 12.5 kg; CDAI: 55 ± 47). The mean baseline peak oxygen consumption (${\dot{\text{V}}\text{O}}_{2\text{peak}}$) were 27.3 ± 7.7 and 28.7 ± 8.6 ml/kg/min for HIIT and MICT, respectively. Mean recorded baseline peak power output on the CPET was 169 ± 25 W for HIIT and 153 ± 11 W for MICT. When participants were asked for their preference to a specific group allocation prior to randomisation 74% preferred HIIT, 22% MICT, and 4% control.

### Exercise testing data

Table 1 depicts the exercise testing data during the exercise tests at baseline and weeks 4, 8 and 12 for both HIIT and MICT. There was a significant interaction between time and condition for peak power (F_(3,60)_ = 5.27; *p* <0.01; η_p_^2^ = 0.21). There was a significant effect of time on peak power for HIIT (F_(3,27)_ = 27.81; *p* <0.01; η_p_^2^ = 0.76). For HIIT peak power significantly increased from baseline to week 4 (mean ± SD, 20.5 ± 10.8W, *p* = 0.03). Peak power was not significantly different between weeks 4 and 8 (mean difference ± SE; 10.00 ±8.94W, *p* = 0.31), but power was significantly greater in week 12 compared to 4 (mean difference ± SE; 12.30 ±6.32, *p* = 0.02). There was no significant difference between weeks 8 and 12 (mean difference ± SE; 2.30 ±7.16W, *p* = 1.00). There was no significant effect of time on peak power for MICT (F_(1.69,18.56)_ = 3.62; *p* = 0.05; η_p_^2^ = 0.25). There were no statistically significant differences between HIIT and MICT for peak power at baseline, weeks 4, 8 and 12 (*p* >0.05). Peak HR was similar between conditions throughout the training with no main effect for condition (F_(1,19)_ = 0.09; *p* = 0.77; η_p_^2^ = 0.01) and no main effect for time (F_(3,57)_ = 0.35; *p* = 0.79; η_p_^2^ = 0.02) nor an interaction between time and training (F_(3,57)_ = 1.16; *p* = 0.33; η_p_^2^ = 0.06).

**Table 1:** Mean ± SD peak power and HR during the exercise tests at baseline and weeks 4, 8 and 12 for HIIT and MICT.

	HIIT*				MICT			
	Baseline	4	8	12	Baseline	4	8	12
Peak Power (W)	169 ± 25	190 ± 35^#^	203 ± 29^#^	203 ± 35^#^^$^	153 ± 11	163 ± 36	165 ± 37	165 ± 30
HR (bpm)	181 ± 12	181 ± 10	183 ± 11	179 ± 13	173 ± 11	177 ± 11	176 ± 9	177 ± 9

*significant difference from MICT (p <0.05).

^#^significant difference from baseline.

^$^significant difference from 4 weeks.

### Exercise training data

#### Attendance

Participants attended 62% of HIIT sessions offered and 75% of MICT sessions. The median (range) number of sessions attended was 25 (0–36) and 25 (18–34) for the HIIT and MICT groups, respectively. Eight (62%) of the HIIT participants and eight (67%) of the MICT participants achieved the pre-specified minimum attendance criterion of at least 24 sessions. Two HIIT participants did not attend a single exercise session: one due to illness, and the other due to work and holiday commitments. Another HIIT participant withdrew from the intervention after completing 5 sessions due to moving abroad. The main reasons for sessions being missed were work commitments (25%, 72/286), illness (25%, 71/286 [only two of which were CD-related]) and holiday (14%, 40/286) (data from both exercise groups combined).

### Heart rate responses

**Fig 1:**
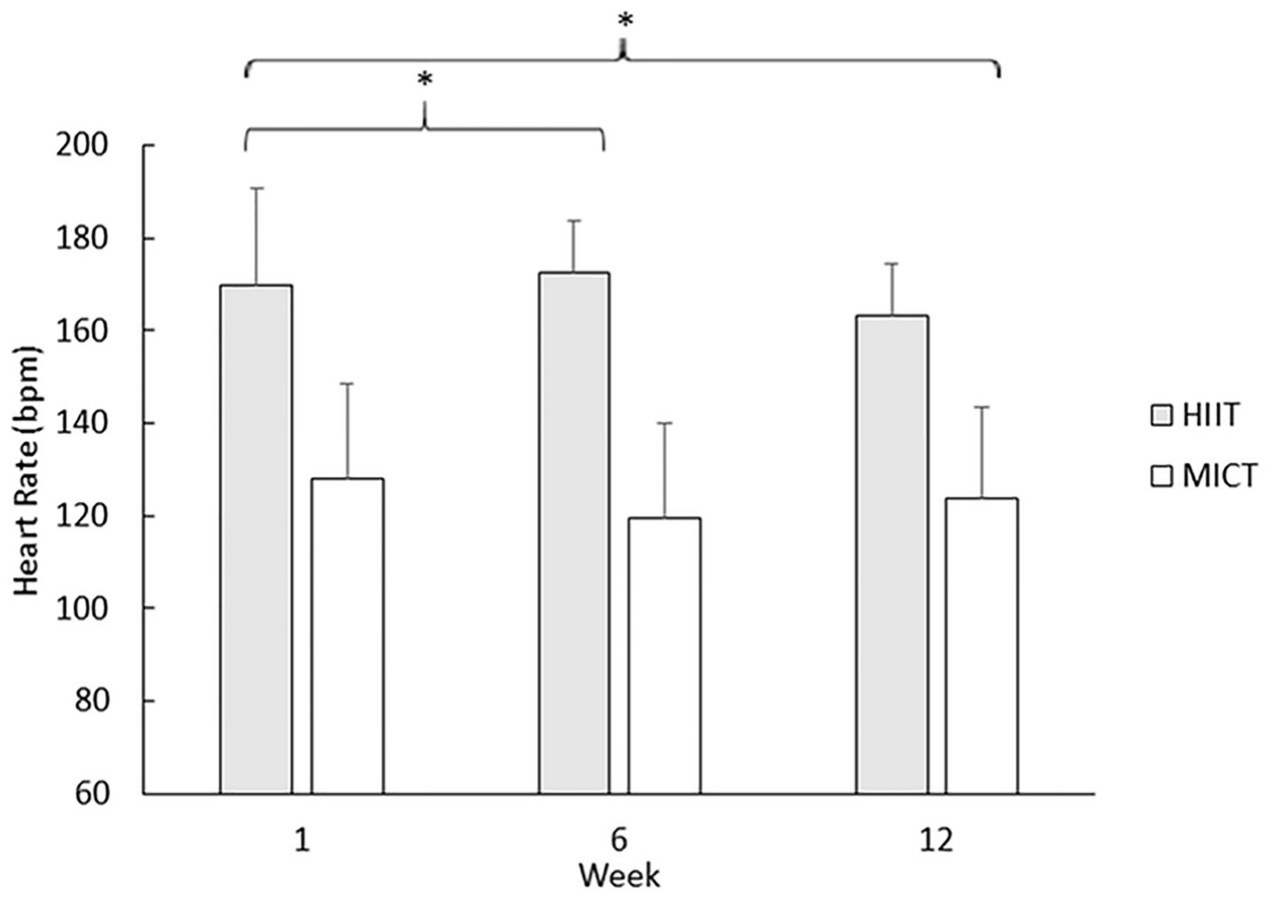
Mean (SD) HR at week 1, 6 and 12. * denotes significant difference from week 1 (*p* <0.05).

There was no significant interaction between time and condition for HR during training (F_(2,26)_ = 0.12; *p* = 0.89; η_p_^2^ = 0.01; Fig 1). There was a main effect of time (F_(2,26)_ = 9.60; *p* <0.01; η_p_^2^ = 0.43) which showed HR to be significantly lower at week 6 (149 ± 6 bpm) and week 12 (148 ± 6 bpm) compared to 1 (155 ± 8 bpm; p = 0.03 and *p* <0.01, respectively). However, there was no significant difference between weeks 6 and 12 (1 ± 2 bpm, *p* = 1.00). There was also a main effect of condition with HR being significantly higher overall during HIIT (173 ± 8 bpm) compared to MICT (128 ±6 bpm, *p* <0.001).

### RPE and affective responses

**Fig 2:**
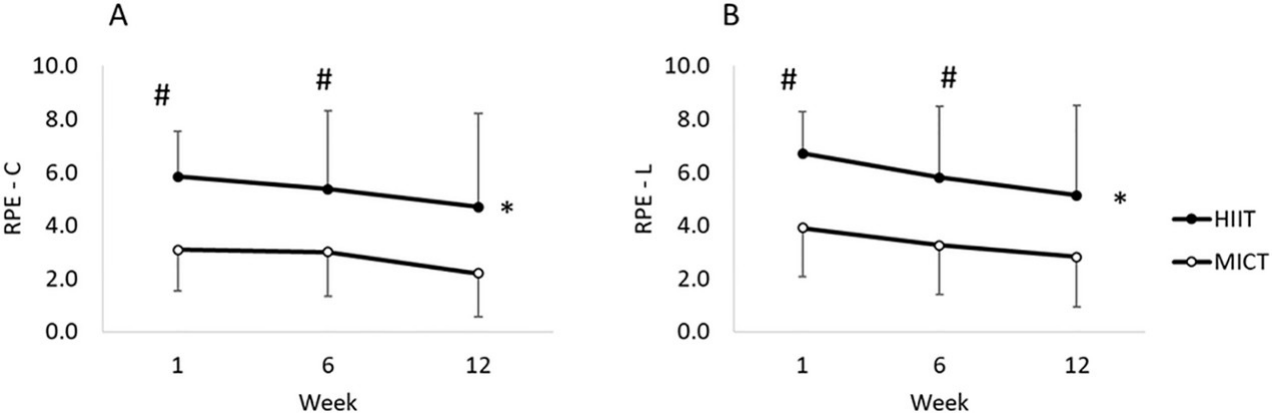
Mean (SD) RPE for central (RPE-C: panel A) and legs (RPE-L: panel B) at week 1, 6 and 12. * denotes significant difference from MICT (*p* <0.05). # denotes significant difference from week 12 (*p* <0.05).

There was no significant interaction for time and condition for RPE-L (F_(2,26)_ = 0.65; *p* = 0.51; η_p_^2^ = 0.05 or RPE-C (F_(2,26)_ = 0.12; *p* = 0.09; η_p_^2^ = 0.01). There was a main effect of condition for RPE-L (F_(1,13)_ = 6.37; *p* = 0.03; η_p_^2^ = 0.33) and RPE-C (F_(1,13)_ = 5.91; *p* = 0.03; η_p_^2^ = 0.31), with RPE-L and RPE-C being significantly greater during HIIT (5.5 ±1.6 and 5.1 ±1.7 i.e. ‘hard’, respectively) compared to MICT (3.3 ± 1.5 and 2.9 ± 1.5 i.e. ‘moderate’, respectively; Fig 2). A main effect of time was demonstrated for both RPE-L (F_(2,26)_ = 7.61; *p* <0.01; η_p_^2^ = 0.37) and RPE-C (F_(2,26)_ = 5.66; *p* = 0.01; η_p_^2^ = 0.30), with values lower at week 12 (3.3 ± 1.7) compared to 1 (4.5 ± 0.76) and 6 (4.1 ± 1.1).

**Fig 3:**
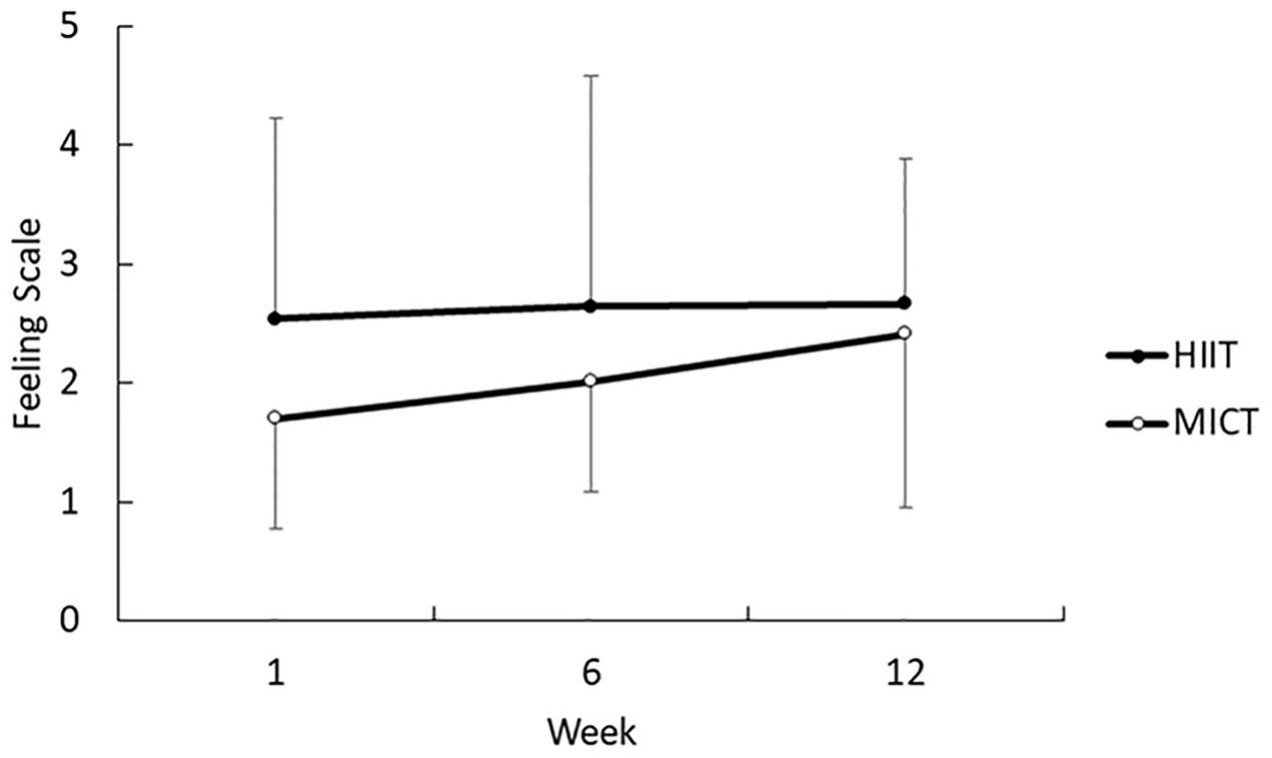
Mean (SD) feeling scale at the end of exercise at weeks 1, 6 and 12.

There was no significant interaction between time and condition for FS (F_(2,26)_ = 1.17; *p* = 0.32; η_p_^2^ = 0.08). There was also no main effect of condition (F_(1,8)_ = 1.51; *p* = 0.25; η_p_^2^ = 0.03) with FS being 2.2 ± 1.9 (i.e. fairly good) for HIIT and 2.1 ± 1.4 (i.e. fairly good) for MICT (Fig 3). Nor was there a main effect of time (F_(3,24)_ = 0.13; *p* = 0.94; η_p_^2^ = 0.04).

### Exercise enjoyment (PACES)

**Fig 4:**
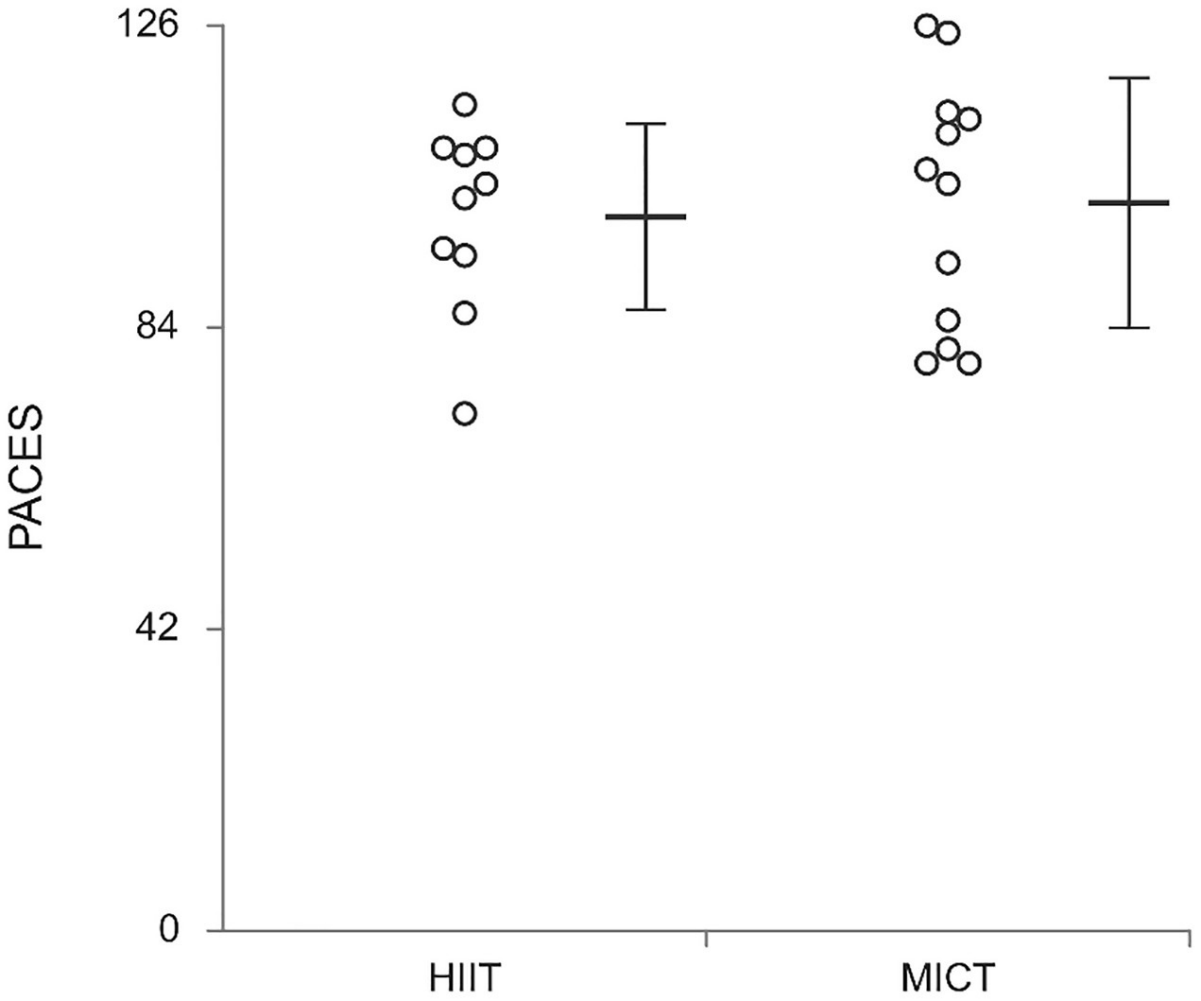
Dot plot of individual exercise enjoyment scores (PACES) and mean (± SD) PACES score post 12 weeks of training for both HIIT and MICT.

**Fig 5:**
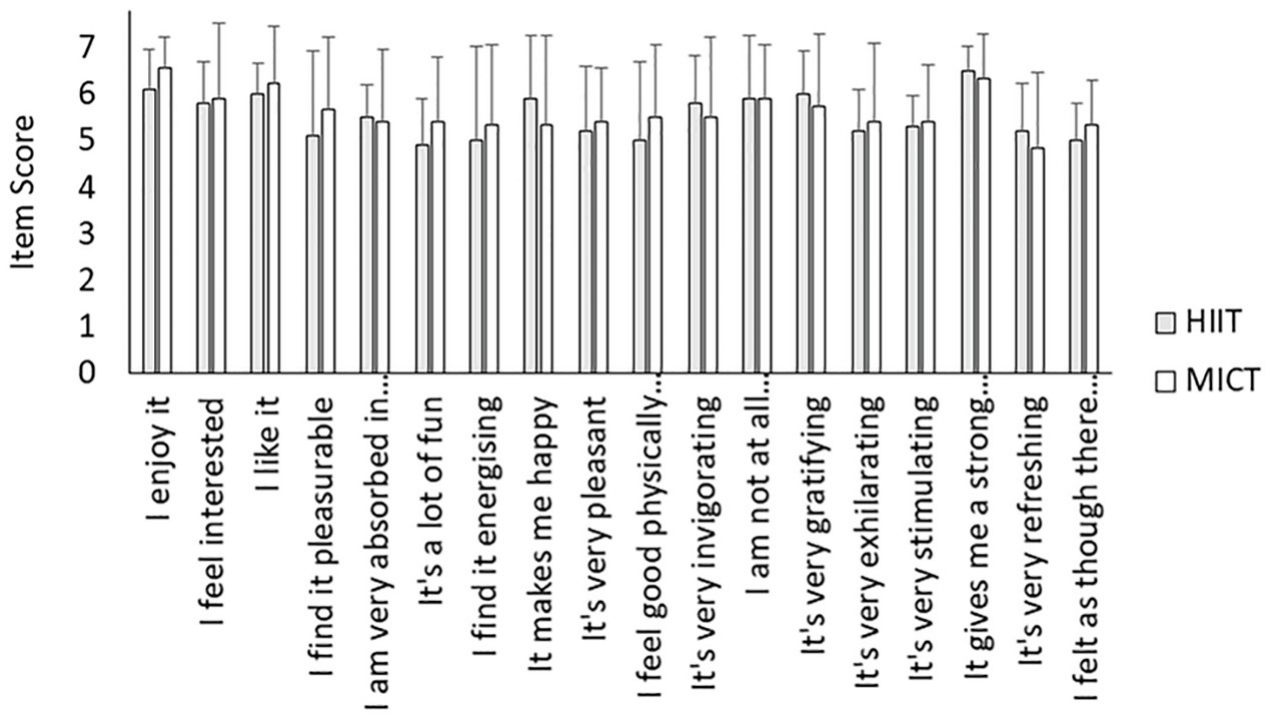
Mean (SD) single item scores (from 1 to 7) for PACES for both HIIT and MICT conditions.

As can be seen in Fig 4 both HIIT and MICT produced high exercise enjoyment scores (mean ± SD 99.4 ± 12.9 and 101.3 ± 17.4, respectively) demonstrating a high level of enjoyment (max score 126), with no significant differences between conditions (t_(20)_ = -0.29; *p* = 0.78; δ = 0.06). When comparing the single item score on PACES between HIIT and MICT (Fig 5) there was no significant interaction between question and condition (F_(5.5,110.8)_ = 0.57; *p* = 0.74; η_p_^2^ = 0.03). There was a main effect of question (F_(5.5,110.8)_ = 3.61; *p* < 0.01; η_p_^2^ = 0.15), but there was no main effect of condition (F_(1,20)_ = 0.08; *p* = 0.78; η_p_^2^ = 0.01).

## Discussion

The present study is the first to compare the enjoyment and affective responses to HIIT and MICT in adults with quiescent or mildly active CD. Despite significantly greater HR and RPE responses during HIIT compared to MICT, these methods of training elicited similar enjoyment and affective responses. Attendance rates were also similar between groups. The enjoyment and affective responses were also generally high, suggesting that adults with quiescent or mildly active CD can find both forms of aerobic exercise training to be acceptable and enjoyable.

Previous research by Tew *et al*. [32] demonstrated that adults with IBD did not meet the physical activity guidelines of the general population. Often a major barrier for participation in physical activity is lack of enjoyment in particular when considering high intensity exercise [15, 20, 21]. As mentioned earlier, high intensity exercise has been suggested to potentially be detrimental to health in adults with IBD due to possible negative side effects, such as gastrointestinal distress [8] which could exacerbate symptoms. Therefore, it was surprising that when participants were asked for their preference to a specific group allocation prior to randomisation 74% preferred HIIT, 22% MICT, and 4% control. This suggests that patients are interested in performing HIIT if they are willing to participate in an exercise clinical trial. As can be seen from the results of this study, not only did participants in both arms of the study enjoy their exercise intervention (either HIIT or MICT) they also felt ‘fairly good’ towards the end of the exercise sessions. We have previously reported that very few exercise-related adverse events occurred during this trial [24]. These results suggest that participants with quiescent or mildly active CD feel ‘fairly good’ when performing both MICT and HIIT.

Along with the barriers to exercise, adhering to an exercise programme can also bring challenges. Demonstrating similar enjoyment between MICT and HIIT suggests both modes of exercise could be employed in a training programme for patients with quiescent or mildly active CD. For participants to maintain a training programme they need to have positive affect responses during exercise as observed in the present study. Mean FS in the present study was 2.2 (SD 1.8; i.e. fairly good) for HIIT and 2.1 (SD 1.3, i.e. fairly good) for MICT at 92.5% of the exercise completed. A meta-analysis by Oliveira *et al*. (19) found that for FS, only six out of 12 comparisons showed beneficial effects for HIIT involving normal weight and overweight-to-obese populations. The authors found no relationship of FS to the fitness characteristics of the participants and therefore suggest that the FS scores were related to the exercise characteristics [19]. When comparing the FS scores in the present study with previous research by Oliveira *et al*. (23) who measured FS pre, every 20% of exercise completed and post exercise for both HIIT and MICT it is apparent that CD patients demonstrated a greater positive FS compared to young healthy adults. The mean ± SD values at the final 20% of exercise in Oliveira *et al*. (23) were -2.7 ± 2.6 for HIIT and 0.8 ± 2.5 for MICT. Differences between our study and Oliveira *et al*. (23) could be a result of the exercise intensity being lower in the present study (90% HRpeak) for 60 seconds compared to 100% VO_2peak_ for 120 seconds. Rhodes and Kates [33] have demonstrated that affective responses during moderate intensity exercise linked positively to future exercise behaviour. Suggesting that participants who feel good during exercise are more likely to continue to perform exercise.

As mentioned previously, HIIT and MICT were matched for energy expenditure although the HIIT main conditioning phase (i.e. excluding warm-up and cool-down) duration was significantly shorter lasting 20 minutes compared to 30 minutes for MICT. HR and RPE were significantly greater for the HIIT sessions compared to the MICT showing a greater exercise intensity. This resulted in greater increases in peak power in the HIIT group compared to the MICT group, suggesting that HIIT could produce greater cardiovascular improvements and ultimately greater cardiometabolic benefits. The exercise intensity and rest intervals of HIIT is important when considering affective and enjoyment responses. There is no universally accepted approach to HIIT and therefore studies often employ different exercise/rest intervals. Research has demonstrated that exercise intervals at 120 seconds produced significantly lower affective responses compared to 60 second intervals [19]. Insufficient rest between intervals can also negatively affect enjoyment and ultimately exercise adherence suggesting a stimulus—recovery ratio of 1:1 produces the best affective responses [19]. The HIIT protocol used in the present study employed a 1:1 ratio of 60 seconds, designed to provide positive affect responses based on research with young sedentary adults [34]. As there are many different methods of HIIT, it may not be appropriate to generalise the findings of the present study to different HIIT approaches e.g. sprint interval training or high volume HIIT (4 minute intervals with recovery of a similar duration) [35].

Mean enjoyment levels based on PACES in the present study were 99.4 (± 12.9) and 101.3 (± 17.4) for HIIT and MICT, respectively. These results are similar in terms of HIIT to Thum *et al*. (15) who found scores of 103.8 ± 9.4 for HIIT (8 x 60s at 85% Wmax followed by 60s at 25% Wmax) in recreationally active participants. In the same study by Thum *et al*. (15) their MICT protocol (20 minutes at 45% Wmax) produced slightly lower levels of enjoyment compared to the present study (84.2 ±19.1). Similarly, Oliveira *et al*. (23) observed values of 97.8 ± 17.3 for HIIT (6 x 120s at 100% VO2peak, 120s at 0% for recovery) and 96.2 ± 16.7 for MICT (20min at 85% VO2peak) in University students. Vella *et al*. [36] observed PACES scores between 74 and 125 for both HIIT (10 x 1 minute at 75–80% of heart rate reserve followed by 1 minute at 35–40%) and MICT (20 minutes at 55–59% of heart rate reserve) in sedentary obese adults. In a clinical population of patients waiting for an elective abdominal aortic aneurysm repair they observed PACES values of 98 (18) with HIIT training (either 8 x 2 minutes or 4 x 4 minutes, 3 x a week for 4 weeks) [37]. Therefore, it appears that patients with quiescent or mildly active CD have similar levels of enjoyment towards both HIIT and MICT exercise in comparison to healthy and clinical adult populations [15, 23, 36, 37].

Previous authors have suggested there is a need to report individual PACES items to signify which items are responsible for high levels of enjoyment [38]. There were no differences in scores for any item of PACES. Each item is scored out of 7, with a higher score demonstrating a greater level of enjoyment. There was a mean score of 5 on every item of the scale for both conditions. To the author’s knowledge the only published study investigating differences on the individual items of the PACES is Malik *et al*. [39] and they found higher levels in items “I got something out of it”, “It’s very exciting” and “It gave me a strong feeling of success” with HIIT compared to MICT. In contrast, the authors observed higher levels of “I feel bored” and “It’s not at all interesting” in the MICT compared to HIIT. They suggest the differences are due to participants perceiving a greater sense of reward, excitement and success following HIIT compared to MICT. The results of the research by Malik *et al*. (39) may differ to the current study due to the fact their participants were aged 12–15 year olds, whereas our study recruited 16–65 year olds.

The study did have some limitations, this was a feasibility trial and as such, with a relatively small sample size, is likely underpowered for these exploratory analyses. Being a small study increases the likelihood of a chance imbalance between groups in the responses to HIIT and MICT throughout the training intervention. Other studies have used a crossover design so participants experience the different exercise protocols, but this is difficult to do when using a 12-week intervention. Another limitation was that enjoyment was only measured at one timepoint. In the study of Heisz et al. [34], PACES was assessed at the end of every week in a 6-week intervention with PACES scores increasing every week in the HIIT arm. However, as the present study measured one time point these changes were not detectable if they existed.

In conclusion, both HIIT and MICT produced high levels of enjoyment as well as feeling ‘fairly good’ suggesting that future exercise trials could include either/both modes as an exercise intervention. One of the major barriers to exercise participation is enjoyment and this study demonstrates that patients with quiescent or mildly active CD appear to enjoy high intensity interval and moderate continuous cycling exercise.
